# Class Address

**Published:** 1873-05

**Authors:** J. B. Ten Eyck


AMERICAN JOURNAL

OF

DENTAL SCIENCE.

Vol. VII. THIRD SERIES--MAY, 1873. No. 1.

### ARTICLE I.

### *Class Address.*

Delivered by J. B. Ten Eyck, M. D., D. D. S., at the Thirty-third Annual Commencement of the Baltimore College of Dental Surgery.

LADIES AND GENTLEMEN: It is with reluctance that I de-[illegible] longer [illegible] the very interesting and instructive address from our esteemed orator, whose words and eloquence are even now lingering with pleasing impressions upon your minds; yet, the members of the graduating class having conferred upon me the honor of representing them, I would ask your kind indulgence for a few moments.

In the weary hours of past labors, have we looked with fond expectations to this happy event, which will mark the "birth-day" on the dial-plate of our professional careers. This happy day with its pleasant associations will never be forgotten, but in the distant future, when the robes with which we to-day have been invested are well worn, we will look back upon it and feel a fresh heart-throb as the whole scene rises up illuminated by the "tender moonlight of memory."

While it is a duty incumbent upon me to pronounce the valedictory salutation to our honored professors and my fellow students, formally severing those familiar relations, which may be numbered among the pleasantest of our lives —to you, my dear friends, I would not breathe a word that could be construed into the language of parting; but, as a compensation for the ties this day severed, we desire to dedicate our lives and profession to your tender care, with a hope of deserving your highest confidence and liberal patronage.

Let us revert for a moment to that period when the beginning of a new era dawned upon the dental profession, the light of which has cast its genial rays far and wide, brightening up the dark corners and dreary wastes of ignorance. At this period which takes us back only about one-third of a century, a handful of men, kindled with a glow of earnest desire to rid our profession of its ignorance and sloth, conceived and founded the Baltimore College of Dental Surgery, the first and oldest institution of the kind that the world ever knew, and which stood for a while alone like a beacon light to the wayfarer. Cotemporary with the founding of this institution, was inaugurated the American Dental Convention, another bright additional light to aid in dispelling the gloom; its rays broke forth like those of the morning sun, causing the good seed to spring up, blossom, and bring forth fruit in good time. Soon followed the *American Journal of Dental Science*, another star of magnitude, which successfully reflected the light of science upon the profession. From these stepping stones, dentistry has rapidly taken rank among the learned professions, and is to-day, though youthful, the most vigorous of the fraternity.

One of the most evident signs of the advancement, worth and popularity of dentistry, is the appeal from the ladies to be taken into the arms of the profession. We heartily receive them, because we know their worth and fidelity; because prestige and honor are carried wherever they go; because we have a weakness towards humoring their wishes; and last but not least, because we love them.

The admission of women into the professional and literary world is an improvement worthy of the spirit and progress of the age. The experiment has proven that the evils involved, are not of such magnitude as many were wont to believe. In fact it has proven a decided success, much to the astonishment of many fathers of science, who have opened their eyes to find that woman's brain *can* comprehend something more than delicate sentiment, poetry and music; and this too without detracting from her feminine grace, or any of the charms of her sex. It is shown that she may become a consistent member of a profession without necessarily adopting Dr. Mary Walker costume, or becoming a corrupt politician. "Women's rights" in the offensive sense of the term, may not even be her ambition, and yet she may thirst for the knowledge of science, without *apparent* manifestations of an unbalanced mind.

The people are aware that while in dentistry there are many members of professional ability who deserve their respect and patronage, there are also many quacks and impostors, who are as leeches in society, sapping the life blood from its members, and the money from their pockets; who think of nothing beyond the dollars, and care nothing beyond self. It is important that the public should know who constitute the pretenders, and who constitute the regular learned profession. This can only be done by legislation. In most countries, the *law* lends its powerful aid to draw the line of demarkation, to protect the professions and the public from imposition and deception; but in this land of boasted liberty and equality, the most ignorant boor hangs out and flaunts to the breeze the badge of our profession—goes in with a bold and insinuating address, and plucks his victim under the cloak of the assumed title of doctor of dentistry. The odium of the mistakes, crimes, and failures of this class, is to a great extent visited upon the heads of the regular profession.

It is gratifying, however, to us and to the people, to know that the day and generation of quackery, this bane of our profession and of society, is fast drawing to a close. It is unworthy of the age, and less consonant with the intelligence and progress of the world. Its foundation is built upon sand, and its frail structure is sure to crumble and fall to the ground. We already see a forshadowing of its fate in the initiative taken by several of the Southern and Western States in the enactment of stringent laws for the suppression of empiric and ignorant practice; this spirit of revolution and self protection is being caught by the people, and charlatans will be made to tremble.

The time is near at hand when the only respectable entrance to the profession will be through the regular collegiate course, and when the law and the people will demand of every dentist his credentials, as they do now of every physician.

Our dear friends, for the many manifestations of kindness you have shown us during the progress of our studies, for your presence here this evening, and for those beautiful floral tributes which speak volumes of good wishes in a language of eloquence and beauty, to you we tender our most grateful thanks.

Gentlemen of the Faculty, we meet you to-day for the last time, in the old and familiar relations of preceptor and student, which has existed so pleasantly during our collegiate course. To your kindness and sympathy are we indebted for the encouragement that has rendered our task less difficult to perform. To your diligent and excellent teaching do we largely owe that success and fruition which to-day gladdens our hearts, and fortifies our strength for future endeavors in the world's great field of enterprise.

Since the last annual commencement of this college, when happy faces, warm hearts, and the sweet incense of flowers filled this hall, one of the beloved members of our faculty (Prof. Bond) has been summoned to a brighter world. While we bow in humble submission to the will of an all-wise Providence, it becomes us to drop a tear to the memory of one who contributed so much to the good of the world, and the progress of science. Although his spirit has returned to the God who gave it, his name will never die. His deeds and his influence survive him, teaching us who remain that the most enduring fame is that won by unremitting diligence, eminent christian examples, and a constant devotion to the advancement of a noble profession.

The revered and honored names of Hayden, Harris, Baxley and Bond, will ever shine in the starry firmament of science, brightly illuminating the pathway of the professional pilgrim. They need no pyramid or monument to perpetuate their memory, "A good deed can never die." It is a possession for all time, caught up by rejoicing angels; it is "syllabeled on tongues of air, by rocks and woods and lonely mountain sides. Gentlemen, a shadow crosses our thoughts when we realize that these are our parting words. The friendly interest you have manifested *to us* during the whole period of our associations has *deeply impressed us*, and we part from you with the same feelings of regard and affection, as we do from our dearest friends. Accept then, gentlemen of the faculty, as we bid you farewell, the warmest assurance of our grateful remembrance.

Fellow Students, we have met here to-day to realize our cherished hopes, to receive from our *alma mater* the reward of long and arduous labors. To-day is placed in our hands a passport, which admits us into the ranks of a profession that is honorable and useful, and which may acquire for us the respect, esteem and gratitude of the community in which we live—a passport to that royal road which has been successfully traveled by many who have walked faithfully in its paths before us, and who are glorified in its compensating gratifications and substantial rewards. Our hearts are light, our step is buoyant, our resolutions strong, and of course *we* expect to reach those resting places; but have we counted the cost? Do we know how weary and footsore we shall become, and how tempting it will be for us to sit down by the wayside before we have reached the prize, which "seemed so near and yet so far?" Do we realize that we have chosen a profession whose march is so rapid that the most resolute and active feel somewhat breathless in trying to keep up with its progress? and yet, our vocation is only consonant with the spirit of the age in which we live. In this day and land of ours, to use the forcible metaphor of Carlisle, "the race of life has become intense; the runners tread upon each other's heels; woe to him who stops to tie his shoe strings." When we consider the emotional excitement, the greedy competition, the ceaseless work which characterizes every profession and every calling in this age of wonder and progress, who of *us* but must be spurred on to keep up with the advance guard of the grand army in its warfarea gainst ignorance, false theories, and undeveloped science, resolved to add something to the common fund by hard study and close observation.

Never before in the history of the world have there been so many avenues opened, and so many facilities for obtaining knowledge in all the various branches of science as to-day. Shall we improve them? Who from among us are to be the heroes of our generation—the great men of the future? We all have within ourselves the magnificent possibilities of life; let us use the talents God has given us, and not "hide them in a napkin."

The imperative to labor is upon us all; but this doom carries a covert blessing under its apparent shadow. Our lives are intended to be active, fruitful, and influential. Let us have a heart to do, and a *will* to accomplish, and we have a power to break through obstacles of habit, time, place or circumstances. What the power of the sun is in the physical universe, is the power of the *will* in our intellectual and moral natures. As without the sun there can be no seed-time and harvest, no seasons, no flowers no beauty in nature to make our hearts glad, so without the *will* there can be no noble actions, no advancement in science, no honor or renown. It was this power of will which served the ambition of Cæsar, Napoleon, and Alexander the great, and carried Harvey, and Jenner, and Kelper, through years of toil and research ere they triumphed.

There is an idea likely to insinuate itself on the minds of men generally, where strength of will and pluck is wanting, that luck in some mysterious way will lead to fame and fortune. Such are idle dreams, which intoxicate the senses and ruin our prospects for life. Luck is said to be an *ignis fatuus* that may lead us to ruin, but not to success. Let us not wait, Micawber like, for something to turn up—things do not turn up in this world unless somebody or something applies the power. Inertia is one of the indispensable laws of matter, and things lie flat where they are, until by some tangible force they are put in motion.

Let us ponder deeply over the life-struggle that is before us, and the nature and extent of all the duties which a faithful response to the claims of the profession demands. Let us remember that as votaries of a liberal profession, the obligations are imperative, to cultivate assiduously its science, and to keep pace with its rapid strides.

Gentlemen, that we may glide safely through the storms of life, and preserve a cheerful and blissfnl composure under its vicissitudes, let us at all times entertain an abiding sense of the providence, presence, and goodness of the great Jehovah. We see his loving kindness evinced in manifold ways; in the language of on eloquent author, "The love of God smiles in the beauty and verdure of spring, and flows brightly in the ripling waters; it sings in the simple and touching melodies of nature, and warbles and rejoices in the shouts of harvest."

Gentlemen of the graduating class, farewell.

